# Critical Step Length as an Indicator of Surface Supersaturation during Crystal Growth from Solution

**DOI:** 10.1021/acs.cgd.1c01249

**Published:** 2022-01-13

**Authors:** Robert Darkins, Ian J. McPherson, Ian J. Ford, Dorothy M. Duffy, Patrick R. Unwin

**Affiliations:** † London Centre for Nanotechnology, University College London, 17-19 Gordon Street, London WC1H 0AH, U.K.; ‡ Department of Chemistry, University of Warwick, Coventry CV4 7AL, U.K.

## Abstract

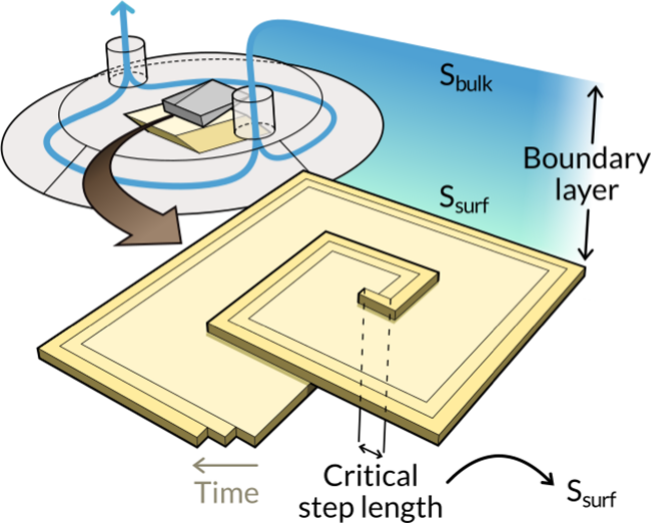

The surface processes that control crystal growth from solution can be probed in real-time by *in situ* microscopy. However, when mass transport (partly) limits growth, the interfacial solution conditions are difficult to determine, precluding quantitative measurement. Here, we demonstrate the use of a thermodynamic feature of crystal surfaces—the critical step length—to convey the local supersaturation, allowing the surface-controlled kinetics to be obtained. Applying this method to atomic force microscopy measurements of calcite, which are shown to fall within the regime of mixed surface/transport control, unites calcite step velocities with the Kossel–Stranski model, resolves disparities between growth rates measured under different mass transport conditions, and reveals why the Gibbs–Thomson effect in calcite departs from classical theory. Our approach expands the scope of *in situ* microscopy by decoupling quantitative measurement from the influence of mass transport.

## Abstract 2

The supersaturation at a crystal/solution interface can be computed from the critical step length measured *in situ*. In this way, the surface-controlled kinetics of calcite are established, and in contrast to the original measurements, they are found to be consistent with classical models of crystal growth.

The surface of a crystal growing from solution can be inspected by *in situ* microscopy to determine the rates and mechanisms of growth,^1−4^ as well as the kinetic and thermodynamic effects of additives.^5−8^ These experimental techniques advance our basic understanding of crystallization and inform our efforts to control it, e.g. in the pursuit of new nanomaterials^9^ and growth inhibitors.^10^ However, *in situ* measurements are difficult to interpret when the solution conditions at the surface of the crystal are unknown. This situation can arise when a crystal depletes the surrounding solutes to produce a concentration gradient with a surface supersaturation that deviates from the bulk value. Under this regime of mixed surface/transport control, the surface processes become sensitive to variables that influence mass transport. For example, the distribution, and therefore the history, of steps across the crystal surface will affect the rate of solute depletion; confinement^11^ and probe geometry^12^ can impact convective transport; impurities can yield kinetic effects with nonmonotonic flow rate dependencies;^13^ and mixed control coupled with stochastic step production can drive kinetic instabilities.^14^ Complicated processes may therefore conceal the surface supersaturation.

In this work, we identify the critical step length (defined below) as a readily measurable surface feature that can immediately reveal the interfacial solution conditions by functioning as a yardstick for the local supersaturation. We demonstrate how this feature can be used to recover the true surface-controlled kinetics of a growing crystal by analyzing atomic force microscopy (AFM) data for calcite (CaCO_3_) published across a series of seminal papers.^1−3^ These particular data were chosen because they include the most comprehensive set of critical length measurements available but also because of the broad importance of calcium carbonate, both in the real world and as a rich model system. While the significance of mass transport is well-established in the context of calcite dissolution,^15^ its importance to calcite growth has hitherto been downplayed by the crystal growth community. It will be shown that flow-through AFM studies of calcite growth are significantly limited by mass transport and that accounting for this effect can bridge the gap between experimental observation and basic models of crystal growth.

*In situ* AFM is usually performed within a fluid cell where a flowing solution replenishes the solutes consumed by the growing crystal. Increasing the flow rate diminishes the concentration boundary layer until the reaction becomes limited by the surface kinetics. In practice, this state of surface control is purportedly achieved once the step velocities have lost their flow rate dependence.^16^ However, owing to the complex hydrodynamics of experimental systems, flow rate dependencies can become too weak to be resolved even far from conditions of surface control.^17−19^ We have demonstrated this with a finite element analysis of the AFM apparatus in refs (1−3), where solutes were transported by convection and diffusion through a fluid cell containing a growing crystal of calcite and a probe (Figure 1a). Flux boundary conditions at the crystal surface approximated the experimental crystal growth rates, and the degree of mass transport to a microscopic scan area on the crystal surface was characterized by a boundary layer thickness δ as a function of the flow rate *u* (δ ≈ 418*u*^–0.27^ μm where *u* is in mL/h, Figure 1b). In the AFM experiments, the step velocities were reported to exhibit flow rate independence across 30 ≲ *u* ≲ 40 mL/h.^16^ For comparison, the surface supersaturation in the model changed by 3% across this flow range, which is too small to be resolved by AFM measurements of the step velocity, and yet the surface supersaturation deviated substantially from the bulk (Figure 1a).

**Figure 1:**
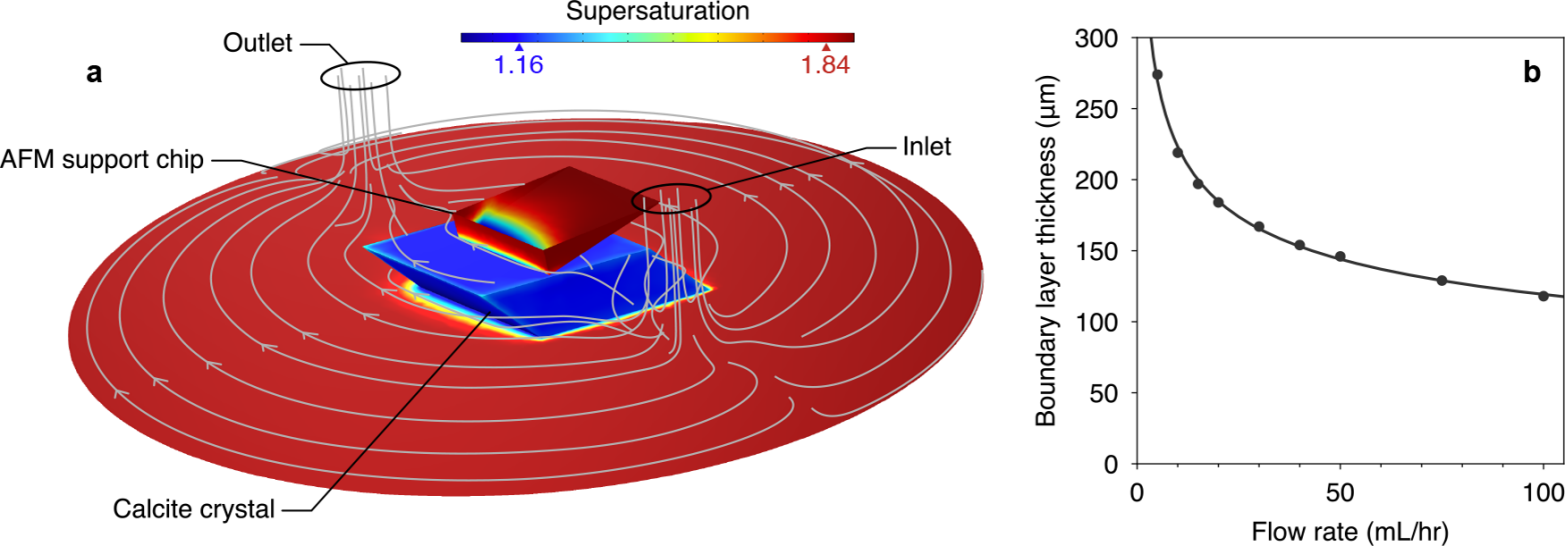
Finite element model of an AFM fluid cell. (a) A rhombohedral calcite crystal (2 mm in width), AFM support chip, and the base of a fluid cell are shown. The colors represent the supersaturation across the surfaces, and the streamlines show the passage of flow between the inlet and outlet. The flow rate is 30 mL/h. (b) Dependence of boundary layer thickness on solution flow rate in the finite element model, evaluated in a region of the crystal surface under the AFM tip (circles). The line is a power law fit.

The true supersaturation at the crystal surface in the AFM experiments can be revealed by examining the surface thermodynamics. A step segment on the surface of a crystal is propelled forward by a chemical potential driving force, where each new ionic row reduces the free energy by an amount proportional to the step length. Resisting this step advance is the free energy cost of extending the length of the two orthogonally adjacent steps. It follows that a finite critical step length *L*_*c*_ exists at which these competing effects balance and the step velocity vanishes,
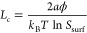
1where *a* = 0.32 nm is the lattice spacing of a single ion in calcite, ϕ is the average step free energy per ion, *k*_*B*_ is Boltzmann’s constant, *T* is the temperature, and *S*_surf_ is the supersaturation at the surface of the crystal. We distinguish *S*_surf_ from the bulk value , where *a*_Ca_ and  are the ion activities in bulk solution and *K*_sp_ is the solubility product of calcite. In crystals that exhibit crystallographically nonequivalent step types, such as the acute and obtuse steps of calcite, each step type will have a distinct stability, and this stability will be reflected in the thermodynamic driving force for growth that underpins *L*_*c*_. In particular, eq 1 will correspond to the critical length of the least stable step type—the obtuse step in the case of calcite.^16^ This perspective of nonequivalent step types conflicts with previous accounts, and we explain it in the Supporting Information.

Critical lengths are manifested at screw dislocations and in the two-dimensional nucleation of islands.^1,20,21^ For calcite, *L*_*c*_ has been directly measured by observing the motion of nascent step segments nucleated at screw dislocations.^1^ However, we focus here on ref (2), where the critical lengths were not directly measured, but where they can be inferred geometrically from the reported step velocities and terrace widths (circles in Figure 2a). For calcite grown under surface control (*S*_surf_ = *S*_bulk_), the critical lengths should follow the blue line in Figure 2a, which is a plot of eq 1 with a step free energy ϕ = 3*k*_*B*_*T*.^22^ However, the experimental results are an order of magnitude too large. This discrepancy between experiment and theory, which has been noted previously,^23^ indicates that the supersaturation at the surface must have been substantially lower than in the bulk, in agreement with our finite element analysis.

**Figure 2:**
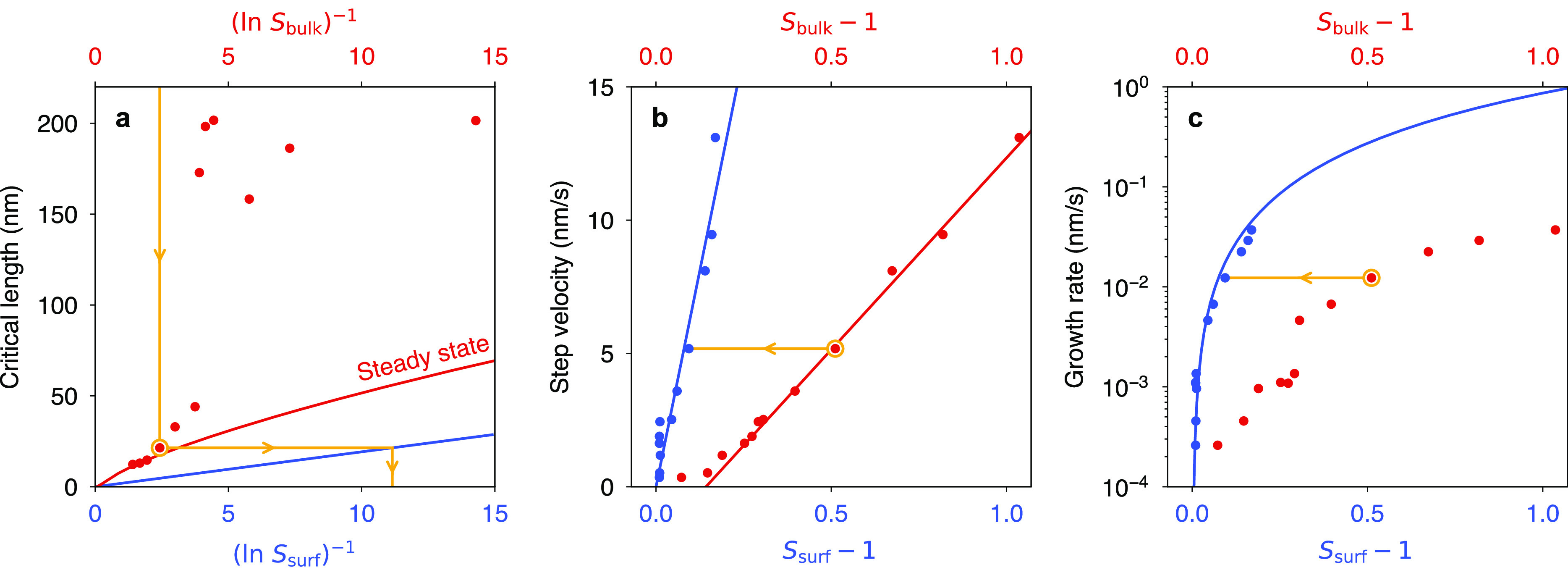
AFM calcite kinetics recalibrated against the surface supersaturation. (a) The theoretical relationship between *L*_*c*_ and *S*_surf_ (eq 1) is shown by the blue line. AFM-derived measurements of *L*_*c*_ (red circles) are larger, reflecting the differences between *S*_bulk_ and *S*_surf_. The measurements are consistent with a steady state correlation (red line) after substantial surface reconstruction, achieved in this case only for large supersaturations. (b) Obtuse step velocities from AFM (red circles) recalibrated against *S*_surf_ (blue circles). The lines are drawn to guide the eye. See the Supporting Information for the acute step velocities. (c) Normal growth rate (red circles) recalibrated against *S*_surf_ (blue circles). The line is a theoretical fit. (a–c) All AFM measurements (circles) have been reproduced from ref (2). The yellow arrows depict, for a single datum, the procedure of using *L*_*c*_ to map from *S*_bulk_ to *S*_surf_.

Once the role of mass transport is recognized, the experimental dependence of *L*_*c*_ on *S*_bulk_ becomes straightforward to interpret. When a crystal is first exposed to a fresh solution, its surface will reconstruct in response. However, it takes time for the new step trains to spread from the dislocation sources across the surface. If sufficient time is allowed for large-scale reconstruction, then *L*_*c*_ will converge to a steady state curve with a dependence on (ln *S*_bulk_)^−1^ that is nonlinear due to the feedback loop between surface structure and *S*_surf_ (red line in Figure 2a); if only partial reconstruction is achieved, then *L*_*c*_ will depend on the stochastic history of the surface (see the low supersaturation points in Figure 2a); and if negligible reconstruction occurs across a series of distinct solution conditions, then *L*_*c*_ will display a linear dependence on (ln *S*_bulk_)^−1^, but the function will be notably offset from the origin such that *L*_*c*_ extrapolates to zero at  (precisely as observed in the direct measurements of *L*_*c*_(1)). See the Supporting Information for a mathematical treatment of these cases.

Irrespective of the surface structure, even far from steady state, the critical length only depends on the prevailing surface supersaturation, and so *S*_surf_ can always be computed from the measured critical length using eq 1. The true saturation state associated with any measurement *x*, such as a step velocity, may also be recovered from *L*_*c*_ as long as the locales of *x* and *L*_*c*_ are similar enough to experience an identical solution environment (we estimate that points within ∼10 μm can be assumed to share a solution environment for AFM studies of calcite). In other words, *L*_*c*_ allows a nominal measurement (*S*_bulk_, *x*) to be decoupled from solute transport and mapped to its surface-controlled analogue (*S*_surf_, *x*). Applying this technique to the kinetic data from ref (2) immediately solves two fundamental problems in calcite kinetics.

First, basic growth theory contends that the step velocities of a Kossel crystal should scale as ∼(*S*_surf_ – 1) at low supersaturation, owing to the statistical independence of attachment and detachment events. This contrasts with the observations of ref (2), which exhibit a nonlinear dependence on *S*_bulk_, with a linear segment that is offset from the saturation point (red circles in Figure 2b). These nonlinearities, discussed further in ref (3), have motivated investigations into non-Kossel kinetic models.^24^ However, recognizing that *S*_surf_ and *S*_bulk_ are different due to mixed surface/transport control, the step velocities can be recalibrated as a function of *S*_surf_, revealing a linear dependence consistent with the Kossel–Stranski model (blue circles in Figure 2b).

Second, a recent microfluidic study^25^ measured the normal growth rate of calcite to be 2 orders of magnitude faster than AFM^2^ under nominally similar conditions. The authors speculated that mass transport may have limited growth in the AFM experiments. Indeed, using the critical length to recalibrate the growth rates against *S*_surf_ yields surface-controlled rates that are 2 orders of magnitude faster (Figure 2c), bringing the AFM and microfluidic measurements to within a factor of ∼4.

Mixed surface/transport control can also have subtle kinetic consequences. The dependence of step velocity *v*(*L*) on the step segment length *L* remains an outstanding problem in basic growth theory.^26^ In extreme cases of mass transport control, as well as for high kink density crystals, the step velocity satisfies the length dependence , which we shall refer to as the Gibbs–Thomson rule.^20^ This rule is widely applied, often beyond the conditions for which it was derived. However, highly polygonal crystals with an interkink spacing comparable to the thermodynamic critical length may fail to satisfy the fluctuation–dissipation theorem, resulting in a critical length much larger than the thermodynamic prediction and, significantly, a step velocity that rises abruptly beyond this kinetically determined critical length.^27^ Calcite has been likened to these extremely low kink density crystals, with the suggestion that it too fails to implement the fluctuation–dissipation theorem.^26,28^ However, we have already shown that the unexpectedly large critical lengths of calcite can be attributed to mixed surface/transport control, and we argue that its abrupt step velocity profile *v*(*L*) can be similarly ascribed. Using kinetic Monte Carlo (KMC) simulation, we have computed *v*(*L*) for a calcite-like Kossel crystal for cases representative of surface control (*S*_surf_ = *S*_bulk_) and mixed surface/transport control (*S*_surf_ ≪ *S*_bulk_). The case of surface control was consistent with the Gibbs–Thomson rule, while mixed control was consistent with AFM to within a trivial normalization error (Figure 3). For calcite, the rapid rise in velocity with step length is therefore a consequence of the low surface supersaturation (large critical length) that characterizes mixed control.

**Figure 3:**
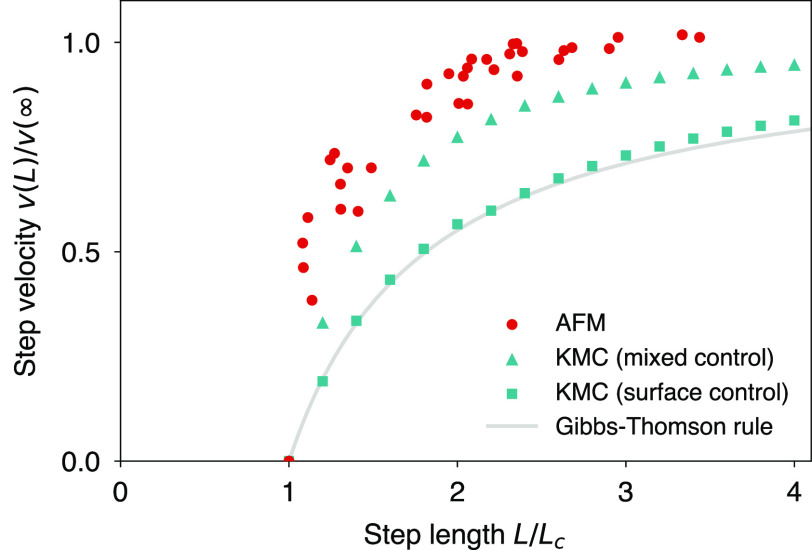
Step velocity dependence on step length. The AFM measurements were taken from ref (1) and span 1.19 ≤ *S*_bulk_ ≤ 1.51. KMC simulations that are representative of surface-controlled growth (*S*_surf_ = *S*_bulk_ = 1.51) are consistent with the Gibbs–Thomson rule, which deviates significantly from AFM. By contrast, KMC simulations that are representative of mixed kinetic control (*S*_surf_ = 1.043, derived from the critical length corresponding to the *S*_bulk_ = 1.51 AFM measurement) are more consistent with the AFM velocity profile.

In conclusion, if the conditions at a crystal/solution interface are unknown, then the information provided by *in situ* microscopy is, at best, underutilized. At worst, the observations can be misinterpreted if surface control is wrongly assumed, e.g. based on the weak dependence of mass transport on flow rate, as we have demonstrated for AFM studies of calcite. In particular, the effects of mass transport can be mistaken for non-Kossel kinetics or a failure of thermodynamics. However, when the critical step length can be established, the transport effects—including the complexities of surface history—can be straightforwardly accommodated. For this reason, we advocate that future *in situ* studies of crystal growth under mixed surface/transport control be accompanied by sufficient data to recover the corresponding critical lengths. In some crystal systems, the critical length would first need to be characterized before our technique could be applied. It is encouraging that many further problems in the field of crystal growth theory may be readily solved upon correcting for mass transport in this way. For example, the dependence of calcite kinetics on solution stoichiometry has proven difficult to reconcile with theory.^29,30^ Significantly, the existing analysis has neglected mass transport, and so this problem is a candidate for a similar treatment.

## Supporting Information Available

Details of the finite element model, the theoretical analysis of AFM kinetics, and the KMC simulations (PDF)
