# Supplementary material for: Critical Step Length as an Indicator of Surface Supersaturation during Crystal Growth from Solution
Source: Cryst Growth Des. 2022 Jan 13;22(2):982–6. doi: 10.1021/acs.cgd.1c01249 (PMC9097158; doi:10.1021/acs.cgd.1c01249)
Supplement: Supplementary file 1 — cg1c01249_si_001.pdf [file cg1c01249_si_001.pdf]

# Supporting information for: Critical step length as an indicator of surface supersaturation during crystal growth from solution

Robert Darkins,† Ian J. McPherson,‡ Ian J. Ford,† Dorothy M. Duffy,† and Patrick R. Unwin*,‡

†*London Centre for Nanotechnology, University College London, 17-19 Gordon Street, London, WC1H 0AH, UK*

‡*Department of Chemistry, University of Warwick, Coventry, CV4 7AL, UK*

E-mail: p.r.unwin@warwick.ac.uk

## Contents

**1 Finite Element Method** **S2**

1.1 Geometry . . . . . . . . . . . . . . . . . . . . . . . . . . . . . . . . . . S2

1.2 Speciation Model . . . . . . . . . . . . . . . . . . . . . . . . . . . . . . S3

1.3 Mass Transport Model . . . . . . . . . . . . . . . . . . . . . . . . . . . S4

1.4 Boundary Layer Thickness . . . . . . . . . . . . . . . . . . . . . . . . . S5

**2 Theoretical Analysis of AFM Data** **S7**

2.1 Critical lengths of nonequivalent step types . . . . . . . . . . . . . . . . . . . S7

2.2 Recovering $L_c$ Geometrically . . . . . . . . . . . . . . . . . . . . . . . . . . . S9

2.3 Acute and Obtuse Step Velocities . . . . . . . . . . . . . . . . . . . . . . . . . S11

2.4 The Dependence of $L_c$ on $S_{\mathrm{bulk}}$ . . . . . . . . . . . . . . . . . . . . . . . . . S12

2.5 Steady State Normal Growth Rate . . . . . . . . . . . . . . . . . . . . . . . . . S15

2.6 Scale of Solution Inhomogeneities . . . . . . . . . . . . . . . . . . . . . . . . . S16

**3 Kinetic Monte Carlo Simulation** **S16**

3.1 Kossel Model of Calcite . . . . . . . . . . . . . . . . . . . . . . . . . . . . . . . S17

3.2 Measuring Step Velocities . . . . . . . . . . . . . . . . . . . . . . . . . . . . . . S17

3.3 Replication of AFM . . . . . . . . . . . . . . . . . . . . . . . . . . . . . . . . . S18

3.4 Model of nonequivalent step types . . . . . . . . . . . . . . . . . . . . . . . . . S19

**References** **S19**

# 1 Finite Element Method

The relationship between the flow rate and the concentration boundary layer established at the surface of a crystal during a typical *in situ* AFM experiment was modelled using the finite element method in COMSOL Multiphysics (v5.6), applying the Transport of Diluted Species and Laminar Flow modules.

## 1.1 Geometry

The AFM experiments[S1] employed a Bruker Nanoscope AFM that features a Digital Instruments flow cell. For the finite element model, we used the Digital Instruments MTFML flow cell geometry from a previous study,[S2] with the addition of a $2 \times 2 \times 0.5$ mm$^3$ rhombohedron

at the origin to represent the calcite crystal. The cantilever was based on the geometry of the probe described in ref.[S1] and was positioned at the surface of the rhombohedron. A $9 \times 9\ \mu m^2$ square patch on the crystal surface, with the AFM tip at the centre, was taken to represent the scan area.

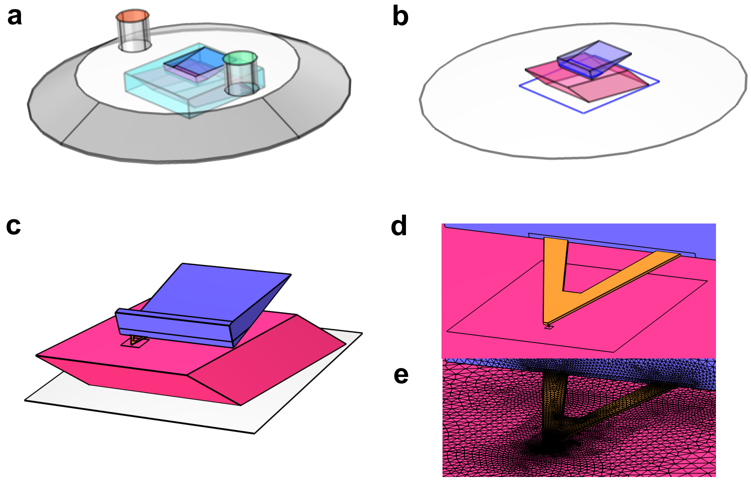


Figure S1: The geometry and mesh considered in the FEM model. (a) The whole cell geometry (top surface removed for clarity), showing the inlet (green), outlet (red) and bounding box for refined model (cyan). (b) View inside the bounding box showing the AFM probe body (without cantilever, blue) and rhombohedron representing calcite (pink). (c) The refined model geometry, with the cantilever (orange) shown enlarged in (d). (e) The mesh density around the cantilever and region of interest.

To accurately simulate the conditions near the cantilever, a multi-scale approach was adopted. A fine mesh of size 500 nm was used within a small domain encompassing the rhombohedral crystal and the cantilever, and a coarser mesh applied elsewhere. The meshes were refined until the solute flux within the region of interest changed by less than 3%.

## 1.2 Speciation Model

The model describes the transport of $Ca^{2+}$ and $CO_3^{2-}$ ions only, where the inlet concentrations were based on the highest-supersaturation experiment in ref.[S1]

In preliminary work, we examined the role of other species ($HCO_3^-$, $NaCO_3^-$, $CaCO_3^0$) on

buffering the local supersaturation. To do this, the initial speciation of the inlet solution was evaluated using the PHREEQC code and database,[S3] and local equilibrium speciation was subsequently maintained throughout the simulated fluid cell. Due to the computational cost of this model, we were limited to a 2D plane parallel to the flow and perpendicular to the surface of the crystal. Initial conditions for the 2D model came from projections of slices of a 3D model that considered the transport of all species but without equilibrium reactions. Good correspondence between the 2D and 3D models, especially close to the crystal surface, gave confidence in this approach. The effect of including equilibrium speciation within the 2D model had only a small ($< 10\%$) effect on the final surface supersaturation, validating the limited species model adopted.

## 1.3 Mass Transport Model

Transport of component $i$ was described by the stationary convection-diffusion equation

$$\nabla \cdot \mathbf{J}_i + \mathbf{w} \cdot \nabla c_i = 0 \tag{S1}$$

where the flux $\mathbf{J}_i$ is determined by the concentration $c_i$ and the diffusion coefficient $D_i$,

$$\mathbf{J}_i = -D_i \nabla c_i \tag{S2}$$

and the solution velocity $\mathbf{w}$ is described by the stationary Navier-Stokes equation and the continuity equation

$$\rho(\mathbf{w} \cdot \nabla)\mathbf{w} = -\nabla p + \mu \nabla^2 \mathbf{w} \tag{S3}$$

$$\nabla \cdot \mathbf{w} = 0 \tag{S4}$$

where $p$ is the pressure, $\rho$ is the density, and $\mu = 8.925528 \times 10^{-4}$ Pa · s is the dynamic viscosity.

A *no flux* condition was applied to all boundaries except:

1. the inlet (the green boundary in Fig. S1a), where the inlet concentrations were fixed at the equilibrium values determined from the speciation model, and
2. the crystal surface (pink boundary in Fig. S1b), where a flux condition was instead applied (see the next section).

A *no slip* condition was applied to all boundaries, except:

1. the inlet, where the solution velocity corresponding to fully developed laminar flow at a given flow rate was specified, and
2. the outlet where the pressure was defined ($p = 0$).

## 1.4 Boundary Layer Thickness

The boundary layer thickness $\delta_i$ associated with species $i$ is defined such that the net flux of solute $i$ from bulk (concentration $c_{\mathrm{bulk},i}$) to the surface (concentration $c_{\mathrm{surf},i}$) is $D_i(c_{\mathrm{bulk},i} - c_{\mathrm{surf},i})/\delta_i$. This net flux must balance with the crystal growth rate $R/\omega$ where $R$ is the normal growth speed and $\omega$ is the molar volume of calcite, resulting in the equation

$$\delta_i = \omega D_i \frac{c_{\mathrm{bulk},i} - c_{\mathrm{surf},i}}{R} \tag{S5}$$

where the average boundary layer $\delta = (\delta_{\mathrm{Ca}^{2+}} + \delta_{\mathrm{CO}_3^{2-}})/2$ is reported in the main text.

To evaluate this parameter under realistic conditions, a flux boundary condition was applied to all faces of the crystal, with a normal flux proportional to $k(S_{\mathrm{surf}} - 1)^2$ for a value $k$ chosen to approximately reproduce the normal growth speed reported experimentally.[S1]

Note that this flux was evaluated locally, producing a non-uniform growth rate across the crystal surface—representative of the experimental crystals which featured an array of screw dislocations across the surfaces.

The boundary layer thickness in the finite element model exhibited a power law dependence $\delta = 418u^{-0.27}$ μm on the flow rate $u$ (in mL/hr). This power law can reveal how the surface supersaturation in an AFM experiment will depend on the flow rate. Suppose the crystal surface structure has fully equilibrated to the solution, and that the normal growth rate of the surface takes the form $R(S_{\mathrm{surf}}) \approx k(S_{\mathrm{surf}} - 1)^2$ (where $k = 1$ nm/s for calcite, see Section 2.5). The experimental step velocities will be proportional to

$$S_{\mathrm{surf}} - 1 = \frac{-1 + \sqrt{1 + 4\xi(S_{\mathrm{bulk}} - 1)}}{2\xi} \tag{S6}$$

which follows from equation (S14), where

$$\xi = \frac{k\delta\gamma}{\omega D K_{\mathrm{sp}}^{1/2}} \tag{S7}$$

and $\gamma$ is the activity coefficient.

In the AFM experiments, the step velocities were reported to plateau across a flow rate $30 \lesssim u \lesssim 40$ mL/hr for nominal supersaturations as high as $S_{\mathrm{bulk}} = 2$.[S4] Using the boundary layer thickness $\delta(u)$ obtained from the finite element model, it follows from equation (S6) that the step velocities will not vary by more than 3% across this flow range (even less at lower supersaturation), consistent with the change observed in the finite element model. By contrast, AFM velocity measurements typically have errors of at least 10%, and suffer from stochastic history-dependence under mixed control if steady state is not fully realised, meaning that this flow rate dependence will be too weak to be resolved.

# 2 Theoretical Analysis of AFM Data

Here we summarise our analysis of the AFM measurements of calcite reported in refs.[S1,S5,S6]

## 2.1 Critical lengths of nonequivalent step types

The geometry of calcite gives rise to two distinct step types, the acute and the obtuse step, which exhibit distinct critical lengths.[S5] At first glance, it is surprising that they have different critical lengths. Each step type has the same pair of adjacent steps (acute on one side, obtuse on the other), and so the free energy cost $\phi$ in equation (1) should ostensibly be the same in both cases. The prevailing explanation for the different critical lengths, first presented in ref.,[S5] is that when a step segment emerges from a screw dislocation, a new corner has to be created. This corner may be curved (due to entropy), and the advancement of the step segment is limited by the cost of creating this curvature. The free energy cost of creating this curved corner will differ for the two step types, and so they will exhibit distinct critical lengths.

However, we believe this explanation is incorrect. A corner becomes curved because a curved corner is a *lower* free energy configuration than a sharp corner. Consequently, the advancement of a step segment will incur a greater free energy cost *after* the curved corner has fully emerged than beforehand. This latter cost will be responsible for the observed critical length, and it will be the same for both step types for the reason outlined above.

An alternative explanation for the different critical lengths comes from considering the different step type stabilities. In the absence of impurities, the velocity of one step type (of infinite length) will never equal zero under the exact same conditions as any other step type (of infinite length), as this would require a remarkable conspiracy between the elementary events associated with each step type. Consequently, as the supersaturation at a crystal/solution interface is decreased, one particular step type—the least stable—will be

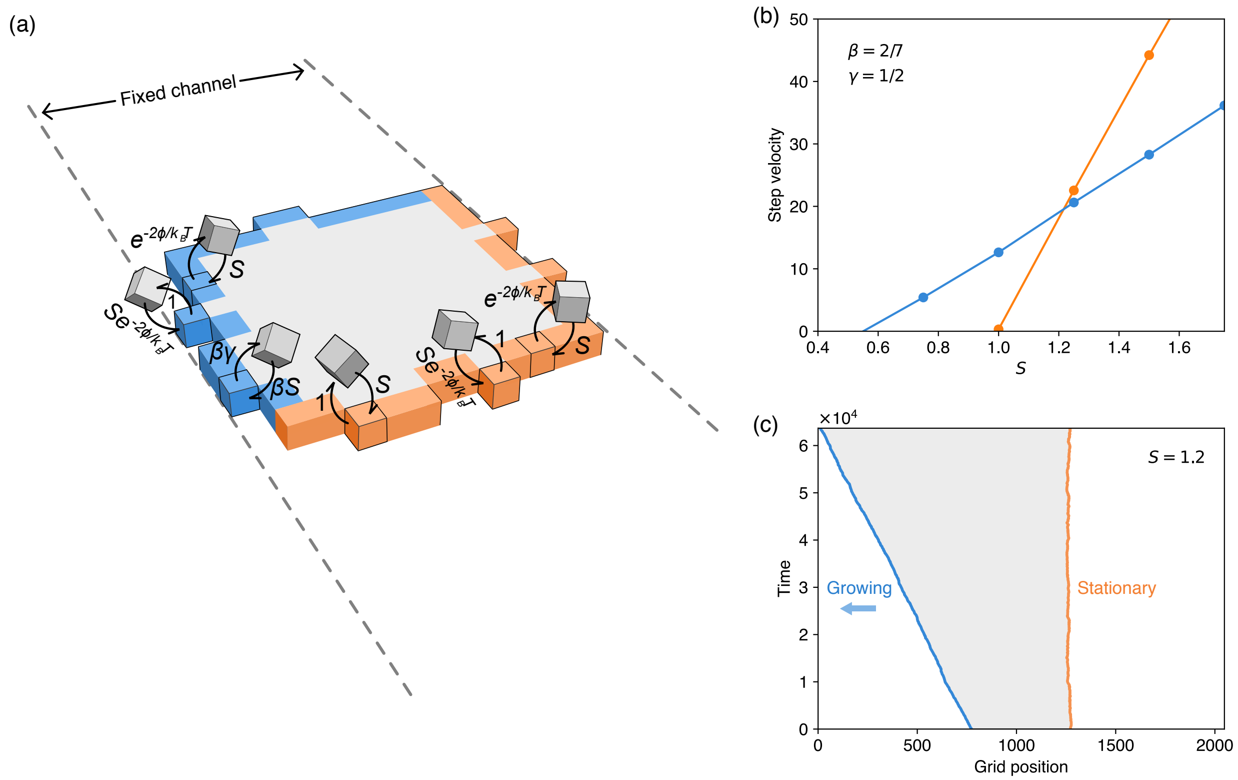


Figure S2: (a) KMC model of a crystal with two nonequivalent step types (orange and blue). The only difference between the step types is the kink flux. (b) The dependence of (infinite) step velocity on $S$ for parameters $\beta = 2/7$, $\gamma = 1/2$. (c) A simulation of an island confined to a channel of finite width (parameters $\beta = 2/7$, $\gamma = 1/2$, $S = 1.2$). The channel width is chosen to be the critical length of the orange step. The blue step nevertheless grows, demonstrating that the step types have distinct critical lengths.

first to halt. For calcite, the least stable step is the obtuse step.[S4] We argue that the stability of a step type must partly determine the corresponding critical length.

To demonstrate this, we have performed a kinetic Monte Carlo simulation (details in Section 3.4) of a simple cubic crystal with two distinct step types, coloured orange and blue in Fig. S2(a). The elementary rate constants have been chosen so that each step type has the same step free energy, but the (infinite) step velocities have different dependencies on the supersaturation (Fig. S2(b)). In particular, the orange step arrests at a higher supersaturation and is therefore less stable than the blue step. In our simulation, we confined an island

to a one-dimensional channel of fixed width. The width was chosen to be the critical length of the orange step. Over the course of the simulation, the orange step fluctuated about its starting position, but the blue step grew (Fig. S2(c)). It follows that the more stable (blue) step has a smaller critical length than the less stable (orange) step, and so the critical length of a step does depend on its stability.

The critical length given by equation (1) in the main text will correspond to the least stable (obtuse) step. To see this, consider a hypothetical crystal that is identical to calcite except the molecular kinetics at the acute step have been modified so that the acute step has the same stability as the obtuse step, but it is modified in a way that preserves its step free energy (this is possible since step free energy and step velocity are independent properties, see Section 3.4). Our claim then follows from three observations:

1. The obtuse step would have the same critical length in both calcite and the hypothetical crystal since the molecular kinetics for the obtuse step and the step free energy for the acute step are identical in both crystals.

2. The hypothetical crystal will have the same solubility as calcite since a crystal (calcite, in this case) ceases to grow once its least stable step (obtuse step) ceases to grow. Both crystals would therefore reach equilibrium under the same conditions.

3. In the hypothetical crystal, the two step types have the same stability (by definition), and so there is no doubt that equation (1) applies.

Note that point 2 in this argument is where the symmetry between the acute and the obtuse step gets broken.

## 2.2 Recovering $L_c$ Geometrically

Critical step lengths associated with a screw dislocation will be geometrically related to the terrace widths and step velocities of the accompanying step outcrop. This relationship will

depend on the crystal geometry.

In the case of calcite, and assuming a unit Burgers vector, the critical length of the obtuse step $L_c$ may be recovered geometrically from the step velocities ($v^{\pm}$) and terrace widths ($\lambda^{\pm}$) of the acute (-) and obtuse (+) steps. First, the average critical length $\langle L_c \rangle$ of the acute and obtuse steps can be computed[S5] via

$$\langle L_c \rangle = \frac{\lambda^{\pm} \sin\theta}{2(1+\frac{v^{\pm}}{v^{\mp}})} \tag{S8}$$

where

$$\theta = \frac{\pi}{2} - \tan^{-1}\frac{0.81(v^{+}-v^{-})}{v^{+}+v^{-}} \tag{S9}$$

measures the angle between adjacent spiral turns.[S6] The obtuse critical length $L_c$ is then related to $\langle L_c \rangle$ by the empirical relationship[S5]

$$L_c \approx 1.167\langle L_c \rangle \tag{S10}$$

The accuracy with which $S_{\mathrm{bulk}}$ can be mapped to $S_{\mathrm{surf}}$ using $L_c$ will depend on how accurately the step free energy $\phi$ is known. The step free energy of calcite has not been measured, but it can be estimated by either the surface free energy or the kink free energy. Measurements for the *surface* free energy include $\phi = 3k_BT$ obtained from nucleation experiments,[S7] and $\phi = 3.2k_BT$ obtained using the vapour adsorption technique.[S8] Estimates for the *kink* free energy can be derived from the observed kink density of calcite[S9] using a model that relates the two; the answer depends on the model used,[S10–S12] but falls between $2.5 \lesssim \phi/k_BT \lesssim 3$. Bringing these various estimates together, we suggest that $\phi = (3 \pm 0.5)k_BT$ is a reasonable measure of the *step* free energy (in more familiar units, $\phi \approx 120 \pm 20$ mJ/m$^2$). The shaded error bars in Fig. S3 show the degree of variation in our theoretical fits to the step velocities that arise from this uncertainty in step free energy.

Significantly, the resulting error in step velocity ($\sim 1$ nm/s) is comparable in magnitude to the errors already present in the AFM velocity measurements.

Note that the data from which $\phi$ has been estimated are unlikely to suffer from the same mass transport distortions that we are ultimately addressing. In the nucleation experiments,[S7] $\phi$ was calculated from nucleation rates which depend on the thermodynamics of critical clusters. However, critical-sized clusters are too small to be limited by diffusion. This can be seen with a back-of-the-envelope calculation: In Section 2.4, we introduce a parameter $\alpha$ to characterise mass transport, and we estimate for calcite that $\alpha \sim 0.1$ for a boundary layer thickness $\delta \sim 100$ µm. It follows from equation (S14) that, for the same surface supersaturation, $\alpha$ will only deviate from 1 (which denotes surface control) when $\delta$ exceeds $\sim 1$ µm. However, the boundary layer of a crystal in a stagnant solution equals the crystal radius, and micron-sized crystals are post-critical as they are observed to be stable in solution. The other experimental data were recorded at equilibrium.

## 2.3 Acute and Obtuse Step Velocities

The step velocities for the acute and obtuse steps, recalibrated against $S_{\mathrm{surf}}$, are both shown in Fig. S3. The solid line was fitted to the obtuse measurements via least squares regression subject to the constraint that the velocity must vanish at $S_{\mathrm{surf}} = 1$, yielding

$$v^{+} = 64.9(S_{\mathrm{surf}} - 1)\ \mathrm{nm/s} \tag{S11}$$

while the dashed line was fitted to the acute measurements without constraint,

$$v^{-} = 16.5(S_{\mathrm{surf}} - 0.9)\ \mathrm{nm/s} \tag{S12}$$

The lower stability of the acute step reported here is consistent with AFM observations.[S4]

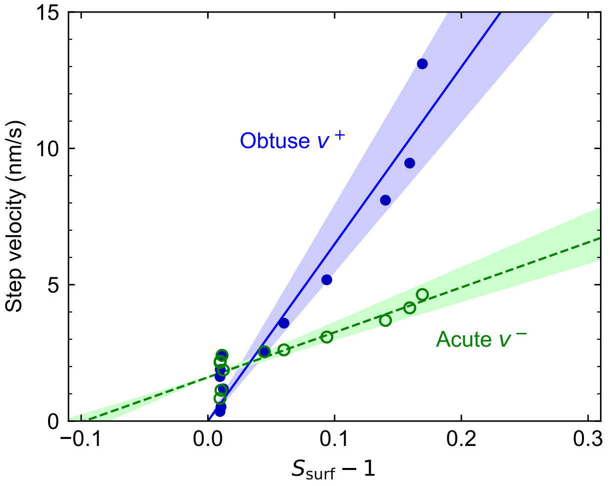


Figure S3: The acute and obtuse step velocities of calcite, measured by AFM[S1,S6] and recalibrated against $S_{\rm surf}$. Least squares regression lines are shown. The shaded error bars correspond to an uncertainty of $\pm 0.5 k_B T$ in the step free energy $\phi$. The acute step is evidently less stable than the obtuse step.

## 2.4 The Dependence of $L_c$ on $S_{\rm bulk}$

The dependence of the critical step length $L_c$ on the surface supersaturation $S_{\rm surf}$ is straightforwardly described by equation (1) in the main text. However, the dependence of $L_c$ on $S_{\rm bulk}$ under mixed surface/transport control will depend on how $S_{\rm surf}$ and $S_{\rm bulk}$ are related, which itself will depend on the prevailing distribution of steps across the surface. To explore this relationship, we consider two limiting cases.

First, suppose $L_c$ is measured against a series of supersaturations $S_{\rm bulk}$ such that the surface is allowed to fully relax and achieve steady state with the solution for each individual measurement. Under this condition, the normal growth rate $R(S_{\rm surf})$ will be the steady state function derived in Section 2.5. Furthermore, we approximate the $Ca^{2+}$ and $CO_3^{2-}$ ions as indistinguishable, with a shared diffusion coefficient $D = (D_{\rm Ca^{2+}} + D_{\rm CO_3^{2-}})/2$ such that the boundary layer thickness (cf. equation (S5)) becomes

$$\delta = \omega D K_{\rm sp}^{1/2} \gamma^{-1} \frac{S_{\rm bulk} - S_{\rm surf}}{R(S_{\rm surf})} \tag{S13}$$

where $\gamma = 0.37$ is the activity coefficient for the AFM experiments[S1] computed from the Davies equation, and $K_{\text{sp}}$ is the solubility product of calcite.

If the boundary layer thickness $\delta$ is known, then $S_{\text{surf}}$ can be recovered for a given $S_{\text{bulk}}$ by numerically solving equation (S13). The critical length $L_c$ then follows from $S_{\text{surf}}$ via equation (1). The solid line in Fig. S4 shows the resulting steady state relationship between $L_c$ and $S_{\text{bulk}}$ for a boundary layer thickness $\delta = 108$ µm, which comes from fitting to the three lowest-$L_c$ data points. We justify this fit on the basis that the $L_c$ measurements vary significantly across the $S_{\text{bulk}}$ range up until these three points where they appear to converge. This is significant since the surface should reconstruct most extensively for these three points since they correspond to the highest supersaturations. Moreover, the resulting functional form is consistent with our theoretical prediction for steady state, and the optimised $\delta$ value is in reasonable agreement with the $\delta \approx 160$ µm obtained from our finite element model. Note that all points that deviate from this line have failed to achieve steady state and are therefore sensitive to their surface histories. Longer equilibration times would have moved each point nearer to the steady state curve.

In the second case, suppose $L_c$ is measured across a range of $S_{\text{bulk}}$, but over a sufficiently short time-frame that the distribution of steps across the surface remains approximately unchanged across all measurements. The significance of not allowing the surface to reconstruct is that it breaks the feedback between step density and surface supersaturation, yielding a characteristic relationship between $L_c$ and $S_{\text{bulk}}$. To analyse this case, it helps to introduce the parameter

$$\alpha := \frac{S_{\text{surf}} - 1}{S_{\text{bulk}} - 1} = \left(1 + \frac{R(S_{\text{surf}})}{S_{\text{surf}} - 1} \frac{\delta\gamma}{\omega D K_{\text{sp}}^{1/2}}\right)^{-1} \in (0, 1] \tag{S14}$$

which follows from equation (S13). This parameter characterises mass transport, with $\alpha = 1$ denoting surface control and a smaller $\alpha$ denoting greater transport control. For a static

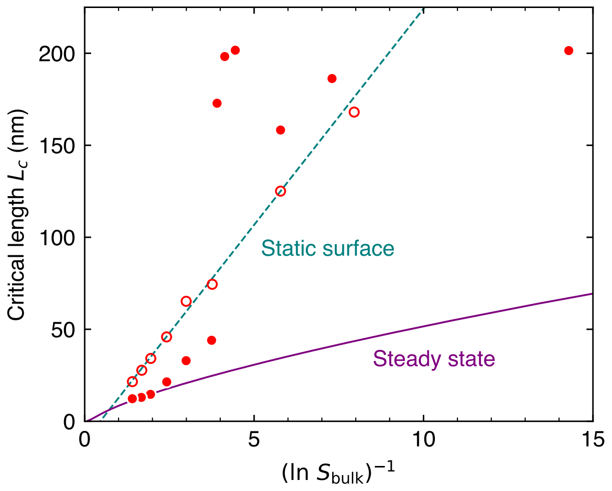


Figure S4: Critical (obtuse) step length dependence on nominal supersaturation. The AFM measurements from ref.[S5] (open circles) are consistent with a static surface (dashed line is a theoretical fit). Three of the AFM measurements from ref.[S1] (closed circles) are consistent with a surface that has achieved steady-state (solid line is a theoretical fit), while the remaining points have achieved only partial equilibration, yielding stochastic history-dependent results.

surface structure, the normal growth rate is linearly proportional to the relative supersaturation, $R(S_{\text{surf}}) \sim (S_{\text{surf}} - 1)$, and so $\alpha$ will be constant with a value determined by the history-dependent surface structure (cf. equation (S14)).

Utilising $\alpha$, the critical length can be shown to simplify to a linear function of $(\ln S_{\text{bulk}})^{-1}$ at low supersaturation,

$$
\begin{aligned}
L_c &= \frac{2a\phi}{k_B T} \frac{1}{\ln S_{\text{surf}}} && \text{(S15)} \\
&= \frac{2a\phi}{k_B T} \frac{1}{\ln(1 + \alpha(\exp(\ln S_{\text{bulk}}) - 1))} && \text{(S16)} \\
&= \frac{2a\phi}{k_B T \alpha \ln S_{\text{bulk}}} \left(1 + \frac{1-\alpha}{2} \ln S_{\text{bulk}} + \ldots\right)^{-1} && \text{(S17)} \\
&\approx \frac{2a\phi}{k_B T \alpha} \left(\frac{1}{\ln S_{\text{bulk}}} - \frac{1-\alpha}{2}\right) && \text{(S18)}
\end{aligned}
$$

where equation (S17) employs a Taylor expansion, and equation (S18) follows from the

binomial approximation. Note that equation (S18) is exact when $\alpha = 1$, as well as in the limit $S_{\mathrm{bulk}} \to 1+$ irrespective of $\alpha$. The largest error occurs when $\alpha \ll 1$ and $S_{\mathrm{bulk}}$ is large. The largest supersaturation sampled in the AFM measurements was $S_{\mathrm{bulk}} = 2$ for which the approximation (equation (S18)) underestimates the critical length by 6% at worst.

The critical length has a larger gradient ($\alpha^{-1}\times$ larger) when plotted against $(\ln S_{\mathrm{bulk}})^{-1}$ (equation (S18)) than when plotted against $(\ln S_{\mathrm{surf}})^{-1}$ (equation (S15)). Moreover, when $L_c$ is plotted against $(\ln S_{\mathrm{bulk}})^{-1}$ it is offset from the origin: $L_c$ is projected to vanish at $\ln(S_{\mathrm{bulk}})^{-1} = \frac{1}{2}(1-\alpha)$ ($\approx \frac{1}{2}$ since $\alpha \ll 1$ under mixed control). Optimising $\alpha = 0.0817$ to fit equation (S18) to the measurements from ref.[S5] reproduces both the gradient and the offset (compare the dashed line to the open circles in Fig. S4), validating this analysis.

## 2.5 Steady State Normal Growth Rate

The normal growth rate of calcite $R(S_{\mathrm{surf}})$ under steady state conditions (where the surface structure has fully equilibrated to the prevailing solution) can be computed as follows.

1. Compute the acute and obtuse step velocities $v^{\pm}$ from equations (S11) and (S12).
2. Compute the obtuse critical length $L_c$ from equation (1).
3. Compute the average critical length $\langle L_c \rangle$ from equation (S10).
4. Compute the terrace widths $\lambda^{\pm}$ from equation (S8).
5. The normal growth rate is then $R(S_{\mathrm{surf}}) = av^{\pm}/\lambda^{\pm}$

The resulting function is plotted in Fig. 2c of the main text (solid line). Note that the growth rate that results from this procedure can be approximated by

$$R(S_{\mathrm{surf}}) \approx k(S_{\mathrm{surf}} - 1)^2, \quad k = 1\,\mathrm{nm/s} \tag{S19}$$

where one ($S_{\text{surf}} - 1$) term comes from the step velocity, and a second comes from the step density via the approximation $\ln S_{\text{surf}} \approx S_{\text{surf}} - 1$.

### 2.6 Scale of Solution Inhomogeneities

Under mixed surface/transport control, a crystal surface with a spatially non-uniform growth rate will induce a spatially non-uniform supersaturation across the surface of the crystal. Acting against these gradients, however, is the lateral diffusion of solutes. The magnitude of solution inhomogeneity across a crystal surface will therefore depend on the balance between the rate of crystal growth and the rate of solute diffusion.

As a crude estimate of this balance, the time-scale over which the steps advance by a monomolecular row (of length $a$) is $\tau = a/v$, and in that window of time, solutes will diffuse a distance $l \sim \sqrt{4D\tau}$ laterally across the surface. Using $a = 0.32$ nm, $v \sim 10$ nm/s, and $D \sim 10^{-9}$ m$^2$/s reveals $l \sim 10$ $\mu$m for calcite. In other words, the solution locally mixes over a scale of $\sim 10$ $\mu$m.

In support of this number, Bracco et al.[S13] reported that calcite step velocity measurements required 20 minutes to equilibrate, during which the steps travelled at an average speed of approximately 10 nm/s. This equates to $\sim 10$ $\mu$m of surface reconstruction before the surface (in the vicinity of a screw dislocation) lost its sense of history.

## 3 Kinetic Monte Carlo Simulation

To study the dependence of step velocity on step length, we developed a simple Kossel model with the same step free energy as calcite. The model was integrated through time using KMC simulation.

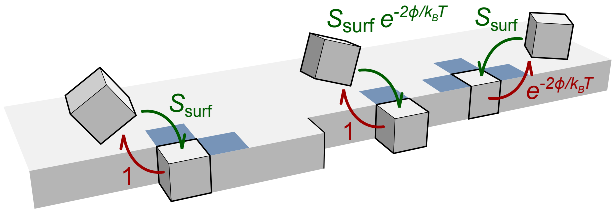


Figure S5: Reaction rates in the Kossel model of calcite. The attachment (green) and detachment (red) rates depend on the number of bonds (shaded squares). Reactions for zero- and four-bonded sites are described in the text.

## 3.1 Kossel Model of Calcite

In our model for a propagating crystal step, the crystal is represented by a two-dimensional grid. Each site is either occupied by an ion or it is not, and has between 0 and 4 (inclusive) neighbouring bonds.

Each site will transition between occupied and unoccupied states at a rate determined by the number of bonds it has, as summarised in Fig. S5. In general, each individual reaction has been constructed so that (i) the creation of a unit of surface induces a free energy cost $\phi = 3k_BT$, and (ii) solute attachment reduces the free energy by $\Delta\mu = k_BT \ln S_{\text{surf}}$. Enforcing the thermodynamic definitions of $S_{\text{surf}}$ and $\phi$ at the level of each individual reaction guarantees that they also hold at the level of the entire step.

As an approximation, we omit this thermodynamic equilibrium for sites with zero and four bonds. In our model, solutes dissolve from zero-neighbour sites at a rate 1 and attach to four-neighbour sites at a rate $S$. In both cases, the reverse reaction should occur at a rate $e^{-4\phi/k_BT}$ to maintain the desired equilibrium. However, since these reverse reactions are rare and occur away from the step edge, we exclude them with the approximation $e^{-4\phi/k_BT} \approx 0$.

## 3.2 Measuring Step Velocities

The crystal configuration is represented on a two-dimensional boolean-valued grid of dimension $w \times h$. The height is $h = 2^{12}$ in all cases, and is terminated at both ends by reflective

boundary conditions. When measuring a step segment of length $L$, the grid width is set to $w = L/a$ with aperiodic boundaries. When estimating the velocity of an (infinite) step, the grid width is set to $w = 2^{10}$ with periodic boundaries.

The crystal initially occupies the area $[0, w) \times [0, h/2)$ so that the reflective boundaries ensure there is a single growth front. The configuration is integrated over time using KMC.[S14] The advancing crystal is periodically displaced back to the centre of the grid (along $y$) to prevent it from exhausting the available grid space.

The system is equilibrated for $10^9$ integration steps, followed by a production period of $10^9$ steps over which the velocity is measured. This routine is repeated 100 times, yielding an average velocity with an aggregate simulation time of $10^{11}$ integration steps, plus a measure of error. The largest standard error associated with any normalised velocity measurement was $\pm 0.18\%$. The resulting KMC measurements (plotted in Fig. 3 of the main text) are normalised with respective to the theoretical critical length $L_c$ (equation (1) in the main text). Note that the step velocity vanishes at the theoretical critical length, as expected.

## 3.3 Replication of AFM

Teng et al.[S5] measured the dependence of step velocity on step length, $v(L)$, across a nominal supersaturation range $1.19 \leq S_{\text{bulk}} \leq 1.51$ using AFM. The resulting velocity profile exhibited no discernible dependence on supersaturation across this range, and is shown in Fig. 3 of the main text.

We computed $v(L)$ using our KMC model for two cases:

1. surface control, where $S_{\text{bulk}} = S_{\text{surf}} = 1.51$, and

2. mixed surface/transport control, where $S_{\text{bulk}} = 1.51$ but $S_{\text{surf}} = 1.043$ in accordance with the experimental critical length $L_c \approx 45.9$ nm that corresponded to this nominal supersaturation (cf. equation (1)).

### 3.4 Model of nonequivalent step types

In Section 2.1, a KMC model of nonequivalent step types was briefly outlined. We elaborate on it here.

The model is a modified version of that previously described (Section 3.1, Fig. S5). Instead of all four step orientations of an island being identical, a pair of steps (blue in Fig. S2) are given distinct elementary rate constants. For both step types (blue and orange), the creation and annihilation of kinks occurs at identical rates, and so they share a step free energy. However, while solutes attach to (detach from) orange kinks at a rate $S$ (1), they attach to (detach from) blue kinks at a rate $\beta S$ ($\beta\gamma$). Consequently, the orange step velocity will scale as $\sim (S-1)$ while the blue step velocity will approximately scale as $\sim \beta(S-\gamma)$. This allows both the magnitude of the step velocity and the stability of the step to be adjusted. For the simulation in Fig. S2(c), we chose $\beta = 2/7$, $\gamma = 1/2$, $S = 1.2$ so that the two step types have the same (infinite) step velocity, demonstrating that their different velocities within the channel result from their distinct length-dependencies, i.e. their distinct critical lengths.

## References


(S1) Teng, H. H.; Dove, P. M.; De Yoreo, J. J. Kinetics of calcite growth: surface processes and relationships to macroscopic rate laws. *Geochimica et Cosmochimica Acta* **2000**, *64*, 2255–2266, DOI: 10.1016/S0016-7037(00)00341-0.

(S2) Adobes-Vidal, M.; Shtukenberg, A. G.; Ward, M. D.; Unwin, P. R. Multiscale visualization and quantitative analysis of L-cystine crystal dissolution. *Crystal Growth & Design* **2017**, *17*, 1766–1774, DOI: 10.1021/acs.cgd.6b01760.

(S3) Parkhurst, D. L.; Appelo, C. A. J. *Description of input and examples for PHREEQC*

*version 3: a computer program for speciation, batch-reaction, one-dimensional transport, and inverse geochemical calculations*; 2013; DOI: 10.3133/tm6A43.

(S4) Fan, C.; Chen, J.; Chen, Y.; Ji, J.; Teng, H. H. Relationship between solubility and solubility product: The roles of crystal sizes and crystallographic directions. *Geochimica et Cosmochimica Acta* **2006**, *70*, 3820–3829, DOI: 10.1016/j.gca.2006.06.011.

(S5) Teng, H. H.; Dove, P. M.; Orme, C. A.; De Yoreo, J. J. Thermodynamics of calcite growth: baseline for understanding biomineral formation. *Science* **1998**, *282*, 724–727, DOI: 10.1126/science.282.5389.724.

(S6) Teng, H. H.; Dove, P. M.; DeYoreo, J. J. Reversed calcite morphologies induced by microscopic growth kinetics: insight into biomineralization. *Geochimica et Cosmochimica Acta* **1999**, *63*, 2507–2512, DOI: 10.1016/S0016-7037(99)00103-9.

(S7) Söhnel, O.; Mullin, J. W. Precipitation of calcium carbonate. *Journal of Crystal Growth* **1982**, *60*, 239–250, DOI: 10.1016/0022-0248(82)90095-1.

(S8) Karaseva, O. N.; Lakshtanov, L. Z.; Okhrimenko, D. V.; Belova, D. A.; Generosi, J.; Stipp, S. L. S. Biopolymer control on calcite precipitation. *Crystal Growth & Design* **2018**, *18*, 2972–2985, DOI: 10.1021/acs.cgd.8b00096.

(S9) De Yoreo, J. J.; Zepeda-Ruiz, L. A.; Friddle, R. W.; Qiu, S. R.; Wasylenki, L. E.; Chernov, A. A.; Gilmer, G. H.; Dove, P. M. Rethinking classical crystal growth models through molecular scale insights: consequences of kink-limited kinetics. *Crystal Growth & Design* **2009**, *9*, 5135–5144, DOI: 10.1021/cg900543g.

(S10) Nielsen, L. C.; De Yoreo, J. J.; DePaolo, D. J. General model for calcite growth kinetics in the presence of impurity ions. *Geochimica et Cosmochimica Acta* **2013**, *115*, 100–114, DOI: 10.1016/j.gca.2013.04.001.

(S11) Burton, W. K.; Cabrera, N.; Frank, F. C. The growth of crystals and the equilibrium structure of their surfaces. *Philosophical Transactions of the Royal Society of London. Series A, Mathematical and Physical Sciences* **1951**, *243*, 299–358, DOI: 10.1098/rsta.1951.0006.

(S12) Joswiak, M. N.; Peters, B.; Doherty, M. F. Nonequilibrium kink density from one-dimensional nucleation for step velocity predictions. *Crystal Growth & Design* **2018**, *18*, 723–727, DOI: 10.1021/acs.cgd.7b01092.

(S13) Bracco, J. N.; Grantham, M. C.; Stack, A. G. Calcite growth rates as a function of aqueous calcium-to-carbonate ratio, saturation index, and inhibitor concentration: Insight into the mechanism of reaction and poisoning by strontium. *Crystal Growth & Design* **2012**, *12*, 3540–3548, DOI: 10.1021/cg300350k.

(S14) Voter, A. F. *Radiation Effects in Solids*; Springer, 2007; pp 1–23, DOI: 10.1007/978-1-4020-5295-8_1.
